# Enrichment of minor allele of SNPs and genetic prediction of type 2 diabetes risk in British population

**DOI:** 10.1371/journal.pone.0187644

**Published:** 2017-11-03

**Authors:** Xiaoyun Lei, Shi Huang

**Affiliations:** Laboratory of Medical Genetics, School of Life Sciences, Xiangya Medical School, Central South University, Changsha, Hunan, China; Universita degli Studi di Roma La Sapienza, ITALY

## Abstract

Type 2 diabetes (T2D) is a complex disorder characterized by high blood sugar, insulin resistance, and relative lack of insulin. The collective effects of genome wide minor alleles of common SNPs, or the minor allele content (MAC) in an individual, have been linked with quantitative variations of complex traits and diseases. Here we studied MAC in T2D using previously published SNP datasets and found higher MAC in cases relative to matched controls. A set of 357 SNPs was found to have the best predictive accuracy in a British population. A weighted risk score calculated by using this set produced an area under the curve (AUC) score of 0.86, which is comparable to risk models built by phenotypic markers. These results identify a novel genetic risk element in T2D susceptibility and provide a potentially useful genetic method to identify individuals with high risk of T2D.

## Introduction

Type 2 diabetes (T2D) is a metabolic disorder that is characterized by high blood sugar and insulin resistance [1]. The number of diabetic cases was globally estimated to be 382 million in 2013 and will be 592 million in 2035 [2]. T2D makes up more than 85% of diabetic cases [2]. The heritability of T2D ranges between 20% and 80% [3]. Development of T2D can be delayed or prevented by maintaining healthy lifestyle behaviors [4, 5]. Certain risk genes such as IRS2 have been identified whose dysfunction contributes to the development of T2D in animal models [6, 7].

Efforts to identify susceptibility loci in T2D have mostly involved genome wide association studies (GWAS) and identified a number of T2D risk single nucleotide polymorphisms (SNPs) and related genes [1, 8, 9]. However, they account for only a small fraction of T2D cases and their mechanisms of action remain largely unknown [1].

Common phenotypic risk factors for prediction of T2D are fasting glucose level, body-mass index, high-density lipoprotein cholesterol level, and age. The phenotypic risk factors alone can obtain an area under the curve (AUC) score between 0.75 and 0.9 [4, 10–12]. However, such information cannot be available at birth. Researchers have also examined the use of single-nucleotide polymorphism (SNPs) to predict the risk of T2D [4]. The AUC scores from these studies ranged between 0.54 to 0.68. However, none could predict T2D cases with complete certainty.

Unlike past studies that focused on individual risk SNPs, our recent studies have shown a role for the genome as a whole in affecting complex traits and diseases [13]. If minor alleles (MA) are more deleterious and under more negative selection, an individual should only be able to tolerate a limited number of MAs. MAs could be defined by using the control population in a matched case-control study. By calculating the fraction of MAs in an individual, or MA contents (MAC) defined as the total number of MAs divided by the total number of SNPs examined, one can compare the average MAC scores of cases relative to controls. More details about the MAC concept have been described in previous studies [14, 15]. We have consistently found that MAC scores are on average higher in complex diseases relative to controls, including Parkinson’s disease [15], lung cancer [16], and schizophrenia [17]. We have further found a subset of MAs that could be used to predict ~2% of these diseases. Furthermore, higher MAC scores are linked with lower reproductive fitness in *C*.*elegans* and yeasts and numerous complex traits in model organisms [14].

To better understand the genetic basis of T2D, we here studied the role of MAC in T2D using previously published GWAS datasets involving a genome wide scanning of 400K-900K SNPs and ~8000 individuals of European ancestry.

## Materials and methods

### Datasets description

A British case and control dataset was downloaded from the Wellcome Trust Case Control Consortium (WTCCC) (https://www.wtccc.org.uk) and included 1,999 T2D cases and 3,004 controls scanned for ~500K SNPs [8]. All 5,003 samples were genotyped with the GeneChip 500K Mapping Array Set (Affymetrix chip), which comprises ~500K SNPs, and the majority were common variants (~80% SNPs with minor allele frequency [MAF] > 1%) and not selected for any diseases. The specific description of SNP genotyping process and the chip were described in the original study [8]. We performed principal components analysis (PCA) using GCTA [18] [19] to remove outliers(S1 Table and S1 Fig showing PCA values and plots). PCA is a method widely used [9, 12] for analyzing population genetic background. While the chosen thresholds based on PCA to exclude outliers were somewhat arbitrary in common practice, our priority was to include as many samples as possible when no clear genetic substructure could be found as visually judged from the PCA plot. After filtering out outliers, ~1,600 cases and ~2,500 controls were retained. They were then separated into two equal size subgroups at random: one for training and the other for validation. Training cohort consisted of ~800 cases and ~1,300 controls and validation cohort ~800 cases and ~1,300 controls (Table 1). Training cohort and validation cohort shared no overlapped samples.

**Table 1:** Basic characteristics of samples used in the study.

	Training		Validation
	WTCCC	WTCCC	phs000091
**Cases**	829	820	1,707
**Controls**	1,270	1,279	2,042
**SNPs**	411,165	411,165	703,407

To verify results from the WTCCC dataset, another independent dataset of T2D case and control cohorts was also downloaded from dbGaP (https://www.ncbi.nlm.nih.gov/gap). As for phs000091, even though the number of individuals at its dbGaP page was said to be 3,000 cases of T2D and 3,000 healthy controls, the actual samples available to be downloaded were only 2,680 cases and 3,148 controls, which belonged to two studies of European Americans (EA): Nurses' Health Study (NHS) and Health Professionals' Follow-up Study (HPFS) [20]. After filtering out outliers by PCA, ~1,700 cases and ~2,000 controls were retained (S2 Table and S2 Fig showing PCA values and plots). All phs000091 samples were genotyped using Affymetrix AFFY_6.0 chips, which comprises ~900K SNPs of mostly common variants (~85% SNPs with MAF > 1%) and were not selected for any diseases. The WTCCC dataset and phs000091 dataset shared ~450,000 SNPs. The final two datasets used are shown in Table 1.

### Data cleaning

The methods for quality control were the same as in previous studies [15, 16, 21]. PLINK was used to remove SNPs in Hardy-Weinberg disequilibrium (Chi-squared test *P*-value < 0.0001 in cases or controls), with > 5% missing data, or with MAF > 0.01 [22]. Only autosomal SNPs were used. Overall, these rigorous steps resulted in retaining ~410,000 SNPs from ~490,000 SNPs in the WTCCC dataset, 703,407 SNPs from ~900,000 SNPs in the phs000091 dataset. Samples with > 10% missing SNPs and non-founders were excluded (i.e., only parents were retained in cases where their children were also sampled). The cleaned datasets were detailed in Table 1.

### Statistical analysis

MAF refers to the frequency at which the second most common allele occurs in a given population. MA was defined as an allele with MAF < 0.5 in a control group. MAC of an individual was calculated by dividing the number of MAs by the total number of SNPs examined [15]. A custom script was used to calculate the MAC value of each sample (https://github.com/health1987/dist). For calculating mean MAC differences between cases and controls in WTCCC cohorts, the training dataset was merged with the validation dataset. Mean MAC values were compared by *t* test. A two-tailed *P*-value less than 0.05 was considered to indicate statistical significance.

Linkage disequilibrium (LD) was performed using PLINK for each pair of SNPs in a window of 200kb SNPs; one SNP from the pair was excluded at random if *r*^2^ > 0.4 [22]. To justify this *r*^2^ threshold, we also tested the results at other *r*^2^ levels (i.e. *r*^2^ = 0.05, *r*^2^ = 0.2, *r*^2^ = 0.6 and *r*^2^ = 0.8).

Here our thinking is: MA is minor for one of two reasons, random and overall under more negative selection. If an allele is overall protective rather than pathogenic, it should not be a minor allele.

For WTCCC, since there are only genotypes and case or control status information available to us, we could only compare the average MAC difference in case and control group to examine the role of MAC in T2D. However, for phs000091, we could download some phenotypic information including age, BMI, alcohol intake, family history of T2D and so on. So for this dataset, we further used multivariate logistic regression test to investigate MAC’s role in T2D relative to other risk factors based on R “glm” function.

### Risk prediction model

In order to obtain a best model for risk prediction, SNPs sets at different *P*-values in training dataset were chosen at first among all SNPs studied here. In addition, to avoid overfitting of the prediction model on the training set from which the SNPs set was derived, LD clumping was performed in WTCCC training cohort. Each MA was given a weighted risk score using the beta value from logistic regression test in PLINK [22], as described previously [15, 16, 23]. Note that in this case, the MA status was determined using the combined cohort of both cases and controls in the training dataset. Asymptotic P-value for each SNP was obtained and different sets of SNPs were chosen to create the genetic risk score at different P-value thresholds of <1E-33, <1E-29, <1E-27, <1E-25, <1E-24, <1E-22, <1E-21, <1E-20, <1E-19, <1E-18, <1E-17, <1E-16, <1E-15, <1E-14, <1E-13, <1E-12, <1E-11, <1E-10, <1E-09, <1E-8, <1E-7, <1E-6, <1E-5, <1E-4, <1E-3, <0.01, <0.03, <0.05, <0.07, <0.09, <0.1, <0.3, <0.5, <0.7, <1 and different *r*^2^ levels (*r*^2^ = 0.05, *r*^2^ = 0.2, *r*^2^ = 0.4, *r*^2^ = 0.6, *r*^2^ = 0.8). The formula for calculating genetic risk score is the following:

$$Genetic\;Risk\;Score(GRS)=\sum_{i=1}^{n}{beta}_{SNPi}+0.5*\sum_{j=1}^{m}{beta}_{SNPj}$$ (1)

SNPi represents MAs in homozygous state and SNPj represents MAs in heterozygous state. A custom script was used to calculate the total weighted genetic risk score by summing up the beta of each MA (S1 File).

### Risk prediction evaluation

Two similar but distinct approaches were performed to estimate the predictive power of the prediction models using the British individuals. For the external cross validation, each model’s predictive power was evaluated using the receiver operating characteristic (ROC) curve. The AUC quantifies the overall ability of the model to discriminate between cases and controls. True positive rate (TPR) is the proportion of cases who had a risk score higher than that of any control individual. Then AUC and the TPR were calculated using the “pROC” R package and Prism 6 (Graphpad). Based on different *P*-values in the training cohorts of British samples, 210 (35X6 = 210) models were constructed. AUC and TPR can be obtained for each model in the validation cohort of British samples.

In internal 5-fold cross-validation analysis, the training cohort was randomly partitioned into 5 subgroups. Of these, a single subgroup was retained as the validation data for testing the model, and the remaining 4 subgroups were used as training data. Then, the cross-validation process was repeated 5 times, with each of the K subgroups used exactly once as the validation data. The 5 results were averaged to produce a single estimation. The model (i.e. MA set) performing the best in both external cross validation and internal cross validation was chosen as the final risk prediction model.

Since GRS proposed above is also a sort of polygenic risk score (PRS) [24], assuming the collective effect of many SNPs, we also compared the prediction accuracy with other PRS based methods (such as PRSice) [25]. In addition, for the best risk model, we also used Nagelkerke *R*^2^ to evaluate its performance base on “fmsb” R package, which denotes the variance explained in disease state by the GRS or PRS.

### SNPs annotations of the best model

Based on the above analyses, SNPs in the risk model performing the best were identified. These SNPs were annotated using the software ANNOVAR [26], resulting in the identification of genes associated with these SNPs. We used DAVID [27] to check the disease or traits associated with these genes. The enrichment in the risk SNPs set was compared by chi squared test with a SNPs set chosen at random.

### Risk prediction in other populations

In addition, for the model performing the best in the British populations, its predictive power was also estimated in the other one independent cohort as described above. Our laboratory protocol was deposited in protocols.io website (http://dx.doi.org/10.17504/protocols.io.j7icrke).

## Results

### Enrichment of minor alleles in T2D cases

We used previously published GWAS datasets of T2D case and control cohorts for our studies. The cleaned datasets after removing genetic outliers were described in Table 1 (PCA values and plots are shown in S1 and S2 Tables as well as S1 and S2 Figs). Total number of samples used here is ~8,000 including ~3,400 T2D cases and ~4,600 controls. In each cohort, we used the control datasets for identifying minor alleles, and then calculated the MAC value of each individual in both the case and the control datasets. In calculating MAC, only SNPs with MAF < 0.4 were included, and SNPs with MAF ≥ 0.4 and ≤ 0.5 were not considered in order to be more certain about the MA status.

**Fig 1:**
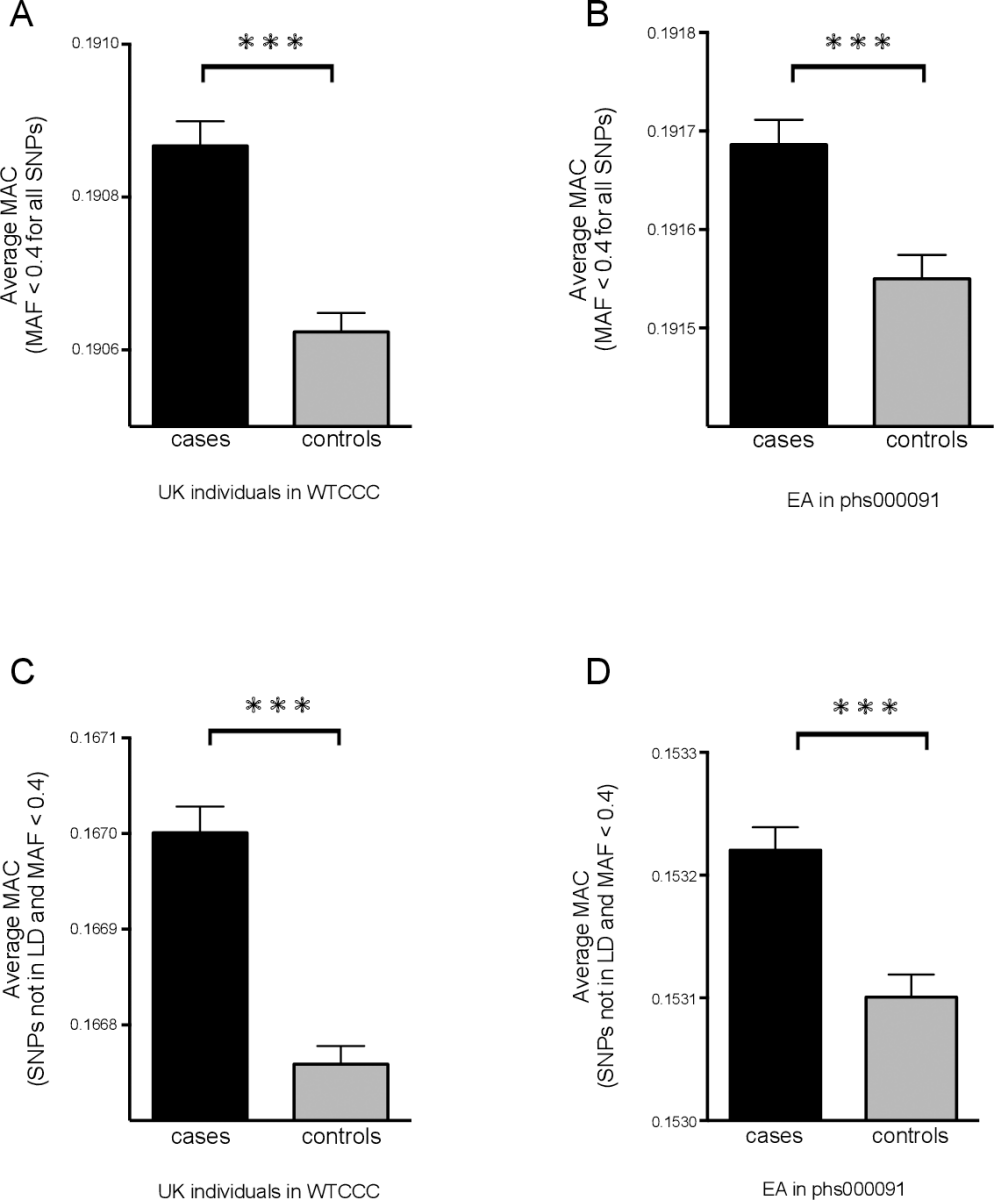
Average MAC (MAF < 0.4) values. Average MAC values of case and control group in UK individuals of European ancestry from WTCCC dataset (A and C) and EA samples from phs000091 dataset (B and D) using SNPs either before (A and B) or after LD clumping (C and D). Student’s t test was used for comparing average MAC. Symbol *** means P value < 0.001.

For British individuals of European origin in the WTCCC study [8], we used the cleaned 340,810 SNPs for the studies here. The average MAC value of the control group was significantly lower than that of the case group (Fig 1A and S3 Table). For 579,767 cleaned SNPs set in the EA dataset phs000091 from dbGaP (Nurses' Health Study and Health Professionals' Follow-up Study), we observed similar result of higher MAC in the cases (Fig 1B and S4 Table). We next analyzed MAC scores using only SNPs that are not in LD at *r*^2^ = 0.05, 0.2, 0.4, 0.6 or 0.8. In British samples from combination of training and validation cohort, ~110,000 autosomal SNPs remained after LD filtering at *r*^2^ = 0.4 and again produced higher average MAC values in cases (Fig 1C and S3 Table). In EA samples of phs000091, ~ 140,000 autosomal SNPs remained after LD filtering at *r*^2^ = 0.4 and also gave higher MAC values in cases (Fig 1D and S4 Table). Similar results were observed at *r*^2^ = 0.05, 0.2, 0.6 and 0.8 (shown in S3 and S4 Tables).

The phenotypic information in phs000091 dataset enabled us to do further analysis comparing MAC with other risk factors. Multivariate logistic regression test is a method used widely for analyses of binary outcome variables such as yes or no disease [28, 29]. If the regression coefficient is positive and the corresponding P value of a variable is lower than 0.05, it might be considered as a risk factor associated with the disease. In the phs000091 case control dataset, we found that MAC, family history, hypertension, high cholesterol, smoking, BMI, age, alcohol intake and heme iron intake all have a positive correlation with the risk of T2D (Table 2). Estimate values (regression coefficients), which represents the effect of a risk factor, indicates MAC effect to be lower than some factors such as BMI and smoking but higher than some others such as physical activity. MAC after LD clumping at different *r*^2^ levels also showed similar results (S5 Table).

**Table 2:** Multivariate logistic regression analyses of T2D in phs000091.

Factors	Explanations	Estimate	SE	P
**MAC**	Minor allele content of all SNPs	0.01046	0.0036	**
**FamdbH**	Family history of diabetes among first degree relatives	1.197	0.0826	***
**Hbp**	Reported high blood pressure at/before blood draw	0.8283	0.08562	***
**Chol**	Reported high blood cholesterol at/before blood draw	0.5204	0.09443	***
**Smk**	Cigarette smoking.	0.28	0.05942	***
**Act**	Total physical activity	-0.002863	0.001401	*
**BMI**	BMI in kg/m2	0.1592	0.009498	***
**Age**	Age in years	0.01201	0.005349	*
**Alcohol**	Alcohol intake in G/day	-0.01058	0.003113	***
**Pufa**	Polyunsaturated fat intake	-0.03732	0.02658	-
**Trans**	Trans fat intake	0.05584	0.07996	-
**Magn**	Magnesium intake in Mg/day	-0.00002306	0.0005684	-
**Ceraf**	Cereal fiber intake in G/day	-0.006541	0.01303	-
**Heme**	Heme iron intake in Mg/day	0.3035	0.08606	***

The multivariate logistic regression was analyzed with R “glm” function. SE denotes standard error.

*** P value < 0.001

** P value < 0.01

* P value < 0.05. P value > 0.05 is indicated by—sign. Where a positive regression coefficient increases the risk of T2D, a negative one decreases the risk of T2D.

### Risk prediction

**Fig 2:**
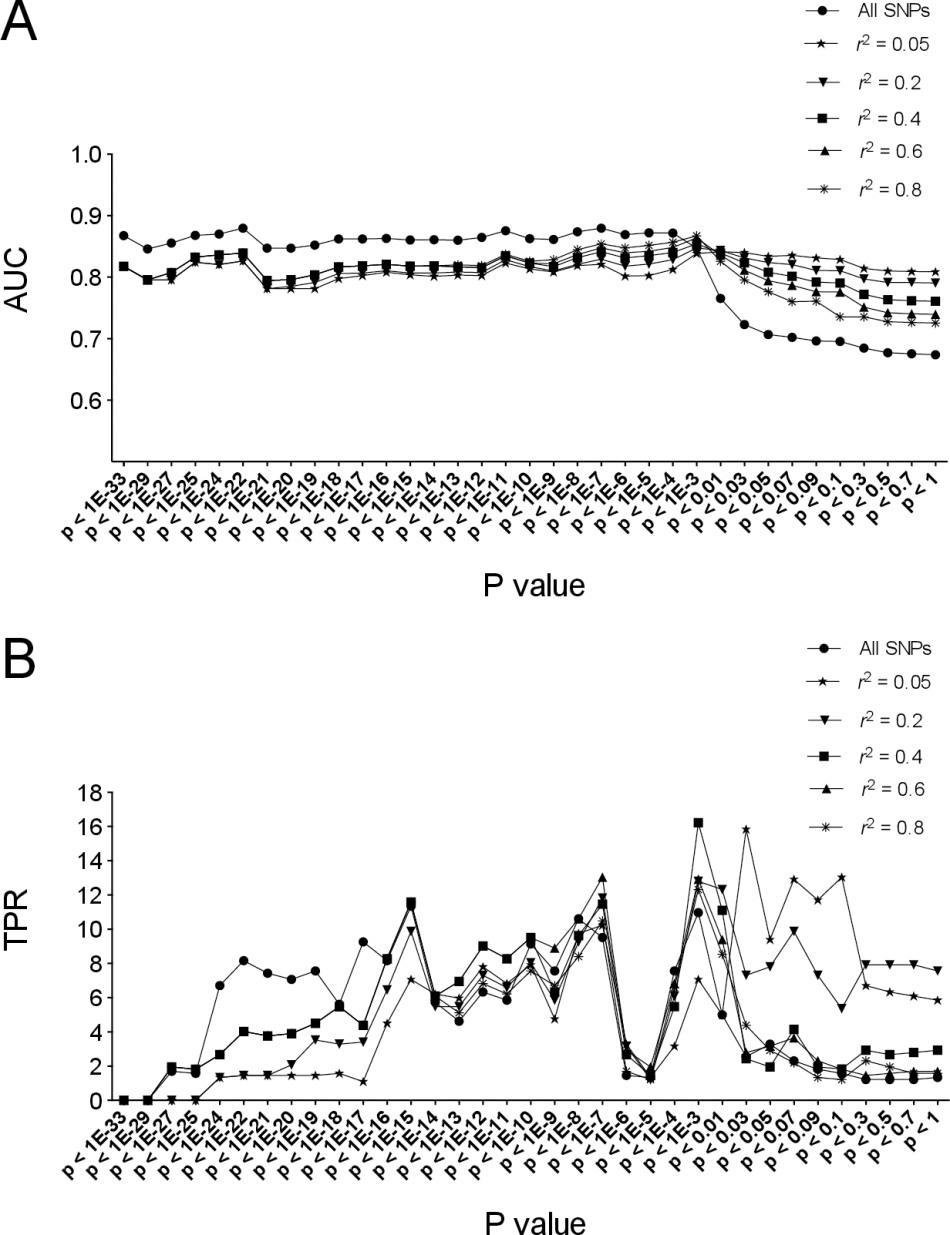
The AUC and TPR values of models in external-cross-validation. Shown are AUC (A) and TPR (B) values of different models consisting of different sets of SNPs at different P values from logistic regression test and different *r*^2^ values at LD clumping.

We aimed to obtain a specific set of MAs from a training dataset (British) that could be used to predict T2D risk for an unrelated dataset (the validation cohort). The training dataset and validation dataset are shown in Table 2. From ~410,000 SNPs after quality control in WTCCC training cohort, ~29,000 autosomal SNPs remained after LD filtering at *r*^2^ = 0.05 (~81,000 loci kept at *r*^2^ = 0.2; 130,000 loci kept at *r*^2^ = 0.4; 180,000 loci kept at *r*^2^ = 0.6; 220,000 loci kept at *r*^2^ = 0.8). In order to obtain an MA set with good prediction performance, 6X35 = 210 models were constructed using different sets of SNPs with different cutoffs of P values from logistic regression tests and different LD *r*^2^ levels. We then used the ROC curve and AUC to examine the predictive power of each set in the external cross validation analyses using the testing dataset (Fig 2, S6 and S7 Tables).

A 5 fold internal cross-validation analysis was performed using the training dataset. Based on external cross validation tests, the model having P-value <0.001 and *r*^2^ = 0.4 was chosen as the best model, which had AUC 0.8545 (95% confidence interval [CI], 0.8378 to 0.8712) and TPR 16.22% (95%CI, 13.76% to 18.92%) in external cross validation test and average AUC 0.8353 and TPR 23.37% in internal cross validation. This model had 363 SNPs, among which 6 loci had minor alleles as defined using the control cohort different from the minor alleles (risk alleles) as defined using the combined population of cases and controls by the PLINK [22] method (see S8 Table for the specific description of the 6 SNPs). When using only the 357 SNPs after removing these 6 SNPs, we obtained slightly improved results with AUC 0.8617 (95% CI, 0.8485 to 0.8780) and TPR 24.56% (95% CI, 19.74% to 25.58%) in external cross validation analysis (see S9 Table for the list of SNPs in this model). The Nagelkerke *R*^2^ of this 357 SNPs set is 0.5084. Thus, including these 6 SNPs in the risk model may worsen the model since their MAF was near 0.5 and hence their minor allele status was not as clean as the rest of the SNPs in the model.

Based on the tool PRSice which is a PRS software, we created 35X5 risk models based on association P value and LD *r*^2^ value (*r*^2^ = 0.05, 0.2, 0.4, 0.6 and 0.8). The best model was a 316 SNPs set at P value <0.001 and 0.4 *r*^2^, which achieved AUC of 0.8563 (95% CI, 0.8397 to 0.8730) and TPR of 20.61% (95% CI, 17.89% to 23.54%). Its Nagelkerke *R*^2^ is 0.4951. So, our method here appears to be comparable or slightly better than the PRS method.

**Fig 3:**
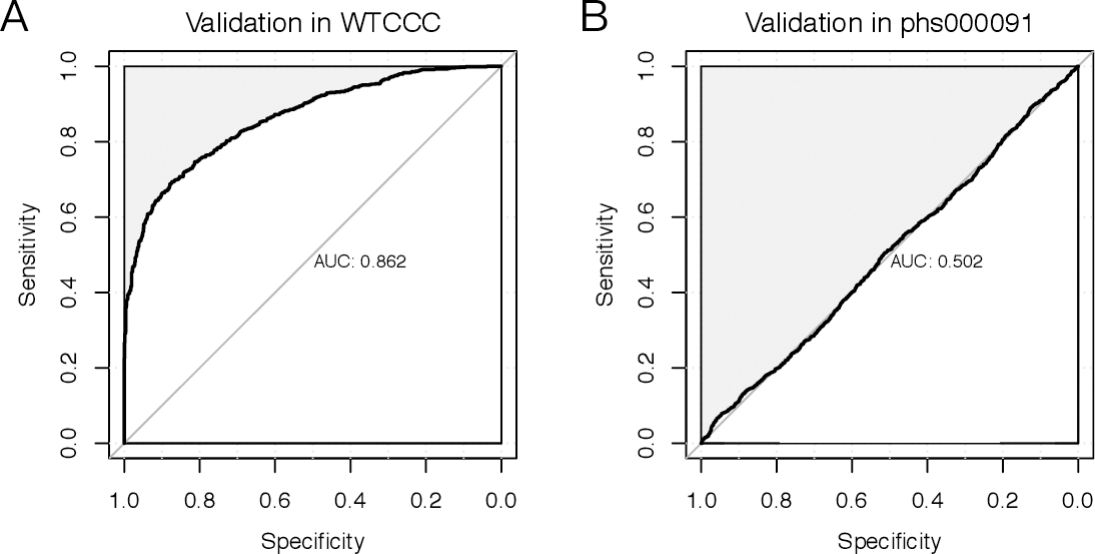
The receiver operating curve for the risk prediction model. (A) WTCCC refers to British population; (B) phs000091 refers the EA samples.

For the 357 SNPs set that performed the best in the British samples (Fig 3A), we further examined it in another dataset phs000091 consisted of EA samples and did not obtain good AUC values (Fig 3B). For the 357 SNPs, there were only 180 loci in phs000091. These results indicate that our model here may only be applicable to British samples, which was expected since different populations should have different MAF in most SNPs.

### SNPs annotations

Compared with previously reported loci in GWAS of T2D [1, 8, 9], all of the 357 SNPs in our best model identified above were newly identified. Using ANNOVAR [26], we identified 494 genes related to these risk SNPs as shown in S10 Table. The majority of risk prediction SNPs are located in non-coding regions. We then used the David model [27] to look for the function of these 494 genes. The first five terms associated with these genes are tobacco use disorder, Body Mass Index (BMI), cholesterol, blood pressure and iron (Table 3). For these five aspects, we also compared the enrichment with 531 genes of a 357 SNPs set chosen at random from the WTCCC dataset. We found that genes associated with SNPs from the risk predition model produced higher fractions in those five terms than those associated with randomly selected 357 SNPs.

**Table 3:** The annotation of genes.

	Genes of 357 SNPs for risk prediction	Genes of 357 SNPs chosen at random	P-value
**Tobacco Use Disorder**	180 (36.44%)	159 (29.94%)	0.03223
**BMI related**	106 (21.46%)	87 (19.59%)	0.04593
**Cholesterol related**	105 (21.26%)	39 (7.34%)	2.72E-10
**Blood Pressure**	33 (6.68%)	0 (0%)	4.18E-09
**Iron**	28 (5.67%)	18 (3.39%)	0.1075
**Others**	42 (8.50%)	228 (42.94%)	< 2.2e-16
**Total**	494 (100%)	531 (100%)	-

P value came from chi square test. BMI, body height and body weight are so closely tied to each other that they are put in a category (i.e. BMI related). Cholesterol, cholesterol HDL and cholesterol LDL are so closely tied to each other that they are put in a category (i.e. cholesterol related).

Active smoking is associated with an increased risk of T2D [30, 31]. BMI is one of the obesity indicators and has been shown to be associated with T2D [32].

Cholesterol-lowering therapy has been suggested for all diabetic individuals who are at sufficiently high risk of vascular events [33].

## Discussion

The result of higher MAC of common variants in T2D cases is a novel finding not expected by known works on human T2D. If most MAs are not related to T2D, the average MAC of cases should not be significantly different from the controls. Thus, enrichment of minor alleles may be involved in the development of T2D. Our finding that MAC of cases is higher than controls is consistent with previous studies on complex diseases, i.e. Parkinson's disease [15], lung cancer [16], and schizophrenia [17]. Comparing the MAC index with other known risk factors of T2D, we further confirmed the role of MAC as a novel risk factor of T2D.

Our study here further strengthens the observation that human genetic diversities are presently at optimum level [13, 15, 16, 34–37]. While it may only take one mutation or a few mutations in major effect genes to cause diseases, it would require the collective effects of many minor effect errors to achieve a similar outcome. Individuals with too many inherited random mutations or MAs may need less degree of other alterations (such as diet related risk factors) to pass the T2D threshold and hence have higher susceptibility to T2D. These studies on MAC are consistent with the recently proposed omnigenic model of complex traits [38].

The method of external-cross-validation has been used in many previous studies where prediction models are constructed in a training dataset and their performance is evaluated in a validation dataset [15, 21, 39]. AUC has been used in many previous studies for gauging performance of prediction models [15, 16, 40]. Our predictive model of T2D appears better than many previous results as indicated by AUC values [5, 10, 11] and achieves a TPR of 24.56% with 100% specificity. It is comparable to risk models built by phenotypic markers. Even though the final model for risk prediction consisted of only 357 SNPs, the actual number of SNPs involved may be much larger since our model used LD-independent SNPs.

After comparing prediction accuracy of the present RGS method with that of the previous PRS method, we observed slightly improved results (AUC: 0.8617 VS 0.8563, TPR: 24.56% VS 20.61%, Nagelkerke *R*^2^: 0.5081 VS 0.4951). That the two methods showed similar performance may not be unexpected given that both are based on the theory of polygenic inheritance for complex diseases. However, the GRS model consisted of 357 SNPs, while the PRS model contained smaller number of SNPs (316), which may account for the slight improvement for the GRS method. The PRS method (PRSice Software) excludes SNPs from transition mutations (A<->T or G<->C), which may decrease its power [25].

We found that the predictive power of our model was population specific. The model was created by using British samples and hence should only work for British samples. This is to be expected since different human groups are known to show group specific SNP profiles. Our finding might be potentially useful for genetic screening of T2D in British subject, before obvious risk factors have developed. In addition, we had tried to create some risk prediction models in dbGaP phs000091 cohorts (data not shown), but the result was relatively poor. The reason may be that even though all phs0000091 samples were European Americans, they might still be a bit more heterogeneous than the UK samples in WTCCC [8].

There are 494 genes associated with these 357 SNPs in our T2D risk prediction model. The first three highly enriched terms associated with these genes were tobacco use disorder, BMI, and cholesterol. Active smoking and exorbitant BMI (i.e. obese) are related to an increased risk of T2D. Cholesterol-lowering therapy may be helpful for T2D patients to manage vascular event. These results indicate a specific functional association of these risk SNPs with T2D, thus validating our MAC method here in uncovering T2D risk alleles.

Most of these risk SNPs are located in intronic or intergenic regions, i.e. non protein-coding region. However, this may not mean that these SNPs are nonfunctional [41]. It might be possible to further improve the method in future studies using larger sample sizes and larger number SNPs.

## Supporting information

S1 Fig PCA plots of WTCCC samples. Principal component values of included subjects: (1) 0 ≤ PC1 ≤ 0.001, (2) -0.0006 ≤ PC2 ≤ 0.0004, (3) -0.0008 ≤ PC2 ≤ 0.0002.
(PDF)

S2 Fig PCA plots of phs000091 samples. Principal component values of included subjects: (1) -0.003 ≤ PC1 ≤ -0.001, (2) -0.0035 ≤ PC2 ≤ 0.0005, (3) 0 ≤ PC3 ≤ 0.01.
(PDF)

S1 Table Principal component values of subjects of WTCCC. (XLSX)

S2 Table Principal component values of subjects of phs000091. (XLSX)

S3 Table Average MAC comparison in WTCCC. MAC comparison of all SNPs and not in LD at different r-squared levels.
(XLSX)

S4 Table Average MAC comparison in phs000091. MAC comparison of all SNPs and not in LD at different r-squared levels.
(XLSX)

S5 Table Multivariate logistic regression. Multivariate logistic regression analyses including MAC after LD of T2D in phs000091.
(XLSX)

S6 Table AUC values. AUC values at different p and LD r-squared values in external-cross validation in UK samples of WTCCC.
(XLSX)

S7 Table TPR values. TPR values (%) at different p and LD r-squared values in external-cross validation in UK samples of WTCCC.
(XLSX)

S8 Table The annotation of the 6 SNPs. The annotation of the 6 SNPs excluded from the risk model.
(XLSX)

S9 Table The best prediction model. The 357 SNPs of the best prediction model for T2D.
(XLSX)

S10 Table The annotation of the 357 SNPs. (XLSX)

S1 File Perl script calculating risk score. (TXT)

## References

[1] Voight BF, Scott LJ, Steinthorsdottir V, Morris AP, Dina C, Welch RP, et al Twelve type 2 diabetes susceptibility loci identified through large-scale association analysis. Nature genetics. 2010;42(7):579–89. Epub 2010/06/29. doi: 10.1038/ng.609 ; PubMed Central PMCID: PMCPMC3080658. 20581827 10.1038/ng.609 PMC3080658

[2] Forouhi NG, Wareham NJ. Epidemiology of diabetes. Medicine. 2014;42(12):698–702. doi: 10.1016/j.mpmed.2014.09.007 25568613 10.1016/j.mpmed.2014.09.007 PMC4282306

[3] Ali O. Genetics of type 2 diabetes. World Journal of Diabetes. 2013;4(4):114 doi: 10.4239/wjd.v4.i4.114 23961321 10.4239/wjd.v4.i4.114 PMC3746083

[4] Meigs JB, Shrader P, Sullivan LM, McAteer JB, Fox CS, Dupuis J, et al Genotype score in addition to common risk factors for prediction of type 2 diabetes. New England Journal of Medicine. 2008;359(21):2208–19. doi: 10.1056/NEJMoa0804742 19020323 10.1056/NEJMoa0804742 PMC2746946

[5] Vassy JL, Meigs JB. Is genetic testing useful to predict type 2 diabetes? Best Practice & Research Clinical Endocrinology & Metabolism. 2012;26(2):189–201. 22498248 10.1016/j.beem.2011.09.002 PMC4070012

[6] Withers DJ, Gutierrez JS, Towery H, Burks DJ, Ren J-M, Previs S, et al Disruption of IRS-2 causes type 2 diabetes in mice. Nature. 1998;391(6670):900–4. doi: 10.1038/36116 9495343 10.1038/36116

[7] Brady MJ. IRS2 takes center stage in the development of type 2 diabetes. The Journal of clinical investigation. 2004;114(7):886–8. doi: 10.1172/JCI23108 15467824 10.1172/JCI23108 PMC518671

[8] Burton PR, Clayton DG, Cardon LR, Craddock N, Deloukas P, Duncanson A, et al Genome-wide association study of 14,000 cases of seven common diseases and 3,000 shared controls. Nature. 2007;447(7145):661–78. doi: 10.1038/nature05911 17554300 10.1038/nature05911 PMC2719288

[9] Fuchsberger C, Flannick J, Teslovich TM, Mahajan A, Agarwala V, Gaulton KJ, et al The genetic architecture of type 2 diabetes. Nature. 2016;536(7614):41 doi: 10.1038/nature18642 27398621 10.1038/nature18642 PMC5034897

[10] Wei B, Hu FB, Shuang R, Ying R, Bowers K, Schisterman EF, et al Predicting Risk of Type 2 Diabetes Mellitus with Genetic Risk Models on the Basis of Established Genome-wide Association Markers: A Systematic Review. American Journal of Epidemiology. 2013;178(8):1197 doi: 10.1093/aje/kwt123 24008910 10.1093/aje/kwt123 PMC3792732

[11] Talmud PJ, Hingorani AD, Cooper JA, Marmot MG, Brunner EJ, Kumari M, et al Utility of genetic and non-genetic risk factors in prediction of type 2 diabetes: Whitehall II prospective cohort study. Bmj. 2010;340(340):b4838. 20075150 10.1136/bmj.b4838 PMC2806945

[12] Talmud PJ, Cooper JA, Morris RW, Dudbridge F, Shah T, Engmann J, et al Sixty-five common genetic variants and prediction of type 2 diabetes. Diabetes. 2015;64(5):1830 doi: 10.2337/db14-1504 25475436 10.2337/db14-1504 PMC4407866

[13] Huang S. New thoughts on an old riddle: What determines genetic diversity within and between species? Genomics. 2016;108(1):3–10. doi: 10.1016/j.ygeno.2016.01.008 26835965 10.1016/j.ygeno.2016.01.008

[14] Yuan DJ, Zhu ZB, Tan XH, Liang J, Zeng C, Zhang JG, et al Scoring the collective effects of SNPs: association of minor alleles with complex traits in model organisms. Science China Life Sciences. 2014;57(9):876–88. doi: 10.1007/s11427-014-4704-4 25104319 10.1007/s11427-014-4704-4

[15] Zhu Z, Yuan D, Luo D, Lu X, Huang S. Enrichment of Minor Alleles of Common SNPs and Improved Risk Prediction for Parkinson's Disease. Plos One. 2015;10(7):e0133421 doi: 10.1371/journal.pone.0133421 26207627 10.1371/journal.pone.0133421 PMC4514478

[16] Xiaoyun Lei DY, Zuobin Zhu, Shi Huang. Collective effects of common SNPs and improved risk prediction in lung cancer. bioRxiv 106864; https://doi.org/10.1101/106864. 2017. 10.1038/s41437-018-0063-4 PMC6221896 29523840

[17] He P, Lei X, Yuan D, Zhu Z, Huang S. Accumulation of minor alleles and risk prediction in schizophrenia. Scientific reports. 2017;7(1):11661 Epub 2017/09/17. doi: 10.1038/s41598-017-12104-0 ; PubMed Central PMCID: PMCPMC5601945. 28916820 10.1038/s41598-017-12104-0 PMC5601945

[18] Tishkoff SA, Reed FA, Friedlaender FR, Ehret C, Ranciaro A, Froment A, et al The genetic structure and history of Africans and African Americans. Science. 2009;324(5930):1035–44. doi: 10.1126/science.1172257 19407144 10.1126/science.1172257 PMC2947357

[19] Yang J, Lee SH, Goddard ME, Visscher PM. GCTA: A Tool for Genome-wide Complex Trait Analysis. American Journal of Human Genetics. 2011;88(1):76 doi: 10.1016/j.ajhg.2010.11.011 21167468 10.1016/j.ajhg.2010.11.011 PMC3014363

[20] Cornelis MC, Qi L, Zhang C, Kraft P, Manson J, Cai T, et al Joint effects of common genetic variants on the risk for type 2 diabetes in U.S. men and women of European ancestry. Annals of internal medicine. 2009;150(8):541–50. Epub 2009/04/22. ; PubMed Central PMCID: PMCPMC3825275. 19380854 10.7326/0003-4819-150-8-200904210-00008 PMC3825275

[21] Kang J, Kugathasan S, Georges M, Zhao H, Cho JH. Improved risk prediction for Crohn's disease with a multi-locus approach. Human Molecular Genetics. 2011;20(12):2435–42. doi: 10.1093/hmg/ddr116 21427131 10.1093/hmg/ddr116 PMC3298027

[22] Purcell S, Neale B, Todd-Brown K, Thomas L, Ferreira MA, Bender D, et al PLINK: A Tool Set for Whole-Genome Association and Population-Based Linkage Analyses. American Journal of Human Genetics. 2007;81(3):559–75. doi: 10.1086/519795 17701901 10.1086/519795 PMC1950838

[23] Okbay A, Beauchamp JP, Fontana MA, Lee JJ, Pers TH, Rietveld CA, et al Genome-wide association study identifies 74 loci associated with educational attainment. Nature. 2016;533(7604):539 doi: 10.1038/nature17671 27225129 10.1038/nature17671 PMC4883595

[24] Purcell SM, Wray NR, Stone JL, Visscher PM, O'Donovan MC, Sullivan PF, et al Common polygenic variation contributes to risk of schizophrenia and bipolar disorder. Nature. 2009;460(7256):748–52. Epub 2009/07/03. doi: 10.1038/nature08185 ; PubMed Central PMCID: PMCPMC3912837. 19571811 10.1038/nature08185 PMC3912837

[25] Euesden J, Lewis CM, O'Reilly PF. PRSice: Polygenic Risk Score software. Bioinformatics (Oxford, England). 2015;31(9):1466–8. Epub 2015/01/01. doi: 10.1093/bioinformatics/btu848 ; PubMed Central PMCID: PMCPMC4410663. 25550326 10.1093/bioinformatics/btu848 PMC4410663

[26] Wang K, Li M, Hakonarson H. ANNOVAR: functional annotation of genetic variants from high-throughput sequencing data. Nucleic Acids Research. 2010;38(16):e164 doi: 10.1093/nar/gkq603 20601685 10.1093/nar/gkq603 PMC2938201

[27] Huang da W, Sherman BT, Lempicki RA. Systematic and integrative analysis of large gene lists using DAVID bioinformatics resources. Nature protocols. 2009;4(1):44–57. Epub 2009/01/10. doi: 10.1038/nprot.2008.211 . 19131956 10.1038/nprot.2008.211

[28] Hosmer DW, Hosmer T, Le Cessie S, Lemeshow S. A comparison of goodness-of-fit tests for the logistic regression model. Statistics in medicine. 1997;16(9):965–80. Epub 1997/05/15. . 9160492 10.1002/(sici)1097-0258(19970515)16:9<965::aid-sim509>3.0.co;2-o

[29] Jones KE, Patel NG, Levy MA, Storeygard A, Balk D, Gittleman JL, et al Global trends in emerging infectious diseases. Nature. 2008;451(7181):990–3. Epub 2008/02/22. doi: 10.1038/nature06536 . 18288193 10.1038/nature06536 PMC5960580

[30] Willi C, Bodenmann P, Ghali WA, Faris PD, Cornuz J. Active smoking and the risk of type 2 diabetes: a systematic review and meta-analysis. Jama. 2007;298(22):2654–64. Epub 2007/12/13. doi: 10.1001/jama.298.22.2654 . 18073361 10.1001/jama.298.22.2654

[31] Kapoor D, Jones TH. Smoking and hormones in health and endocrine disorders. European journal of endocrinology. 2005;152(4):491–9. Epub 2005/04/09. doi: 10.1530/eje.1.01867 . 15817903 10.1530/eje.1.01867

[32] Vazquez G, Duval S, Jacobs DR Jr., Silventoinen K. Comparison of body mass index, waist circumference, and waist/hip ratio in predicting incident diabetes: a meta-analysis. Epidemiologic reviews. 2007;29:115–28. Epub 2007/05/12. doi: 10.1093/epirev/mxm008 . 17494056 10.1093/epirev/mxm008

[33] Kearney PM, Blackwell L, Collins R, Keech A, Simes J, Peto R, et al Efficacy of cholesterol-lowering therapy in 18,686 people with diabetes in 14 randomised trials of statins: a meta-analysis. Lancet (London, England). 2008;371(9607):117–25. Epub 2008/01/15. doi: 10.1016/s0140-6736(08)60104-x . 18191683 10.1016/S0140-6736(08)60104-X

[34] Shi H. The Genetic Equidistance Result of Molecular Evolution is Independent of Mutation Rates. Journal of Computer Science & Systems Biology. 2008;1(1):92. 21976921 10.4172/jcsb.1000009 PMC3184610

[35] Huang S. Inverse relationship between genetic diversity and epigenetic complexity. http://precedings.nature.com/documents/1751/version/2. Nature Precedings. 2009.

[36] Dejian Yuan XL, Yuanyuan Gui, Zuobin Zhu, Dapeng Wang, Jun Yu, Shi Huang. Modern human origins: multiregional evolution of autosomes and East Asia origin of Y and mtDNA. bioRxiv: 101410; http://biorxiv.org/content/early/2017/01/18/101410. 2017.

[37] Jostins L, Barrett JC. Genetic risk prediction in complex disease. Human Molecular Genetics. 2011;20(R2):R182–8. doi: 10.1093/hmg/ddr378 21873261 10.1093/hmg/ddr378 PMC3179379

[38] Boyle EA, Li YI, Pritchard JK. An Expanded View of Complex Traits: From Polygenic to Omnigenic. Cell. 2017;169(7):1177–86. Epub 2017/06/18. doi: 10.1016/j.cell.2017.05.038 . 28622505 10.1016/j.cell.2017.05.038 PMC5536862

[39] Hagenaars SP, Hill WD, Harris SE, Ritchie SJ, Davies G, Liewald DC, et al Genetic prediction of male pattern baldness. PLoS Genet. 2017;13(2):e1006594 Epub 2017/02/15. doi: 10.1371/journal.pgen.1006594 ; PubMed Central PMCID: PMCPMC5308812 following competing interests: IJD and DJP are participants in UK Biobank. 28196072 10.1371/journal.pgen.1006594 PMC5308812

[40] Lyssenko V, Laakso M. Genetic Screening for the Risk of Type 2 Diabetes: Worthless or valuable? Diabetes Care. 2013;36(Supplement_2):S120–S6. 23882036 10.2337/dcS13-2009 PMC3920800

[41] Consortium TEP. An integrated encyclopedia of DNA elements in the human genome. Nature. 2012;489(7414):57–74. doi: 10.1038/nature11247 22955616 10.1038/nature11247 PMC3439153

