# Correction: Enrichment of minor allele of SNPs and genetic prediction of type 2 diabetes risk in British population

**DOI:** 10.1371/journal.pone.0191210

**Published:** 2018-01-09

**Authors:** Xiaoyun Lei, Shi Huang

There are errors in the Funding section. The correct funding information is as follows: This work was supported by the Fundamental Research Funds for the Central Universities of Central South University, the National Natural Science Foundation of China [S.H., Grant 81171880], and the National Basic Research Program of China [S.H., Grant 2011CB51001]. The funders had no role in study design, data collection and analysis, decision to publish, or preparation of the manuscript.
