# Supplementary figures and images for: Enrichment of minor allele of SNPs and genetic prediction of type 2 diabetes risk in British population
Source: PLoS One. 2017 Nov 3;12(11):e0187644. doi: 10.1371/journal.pone.0187644 (PMC5669465; doi:10.1371/journal.pone.0187644)

**A** PCA of raw samples in WTCCC

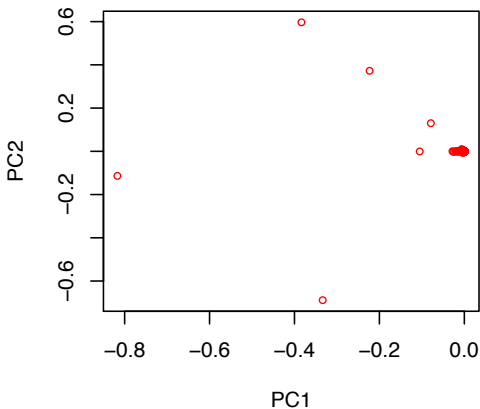

**B** PCA of raw samples in WTCCC

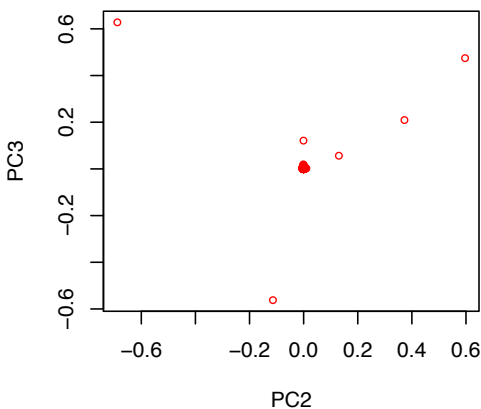

**C** Outliers removed

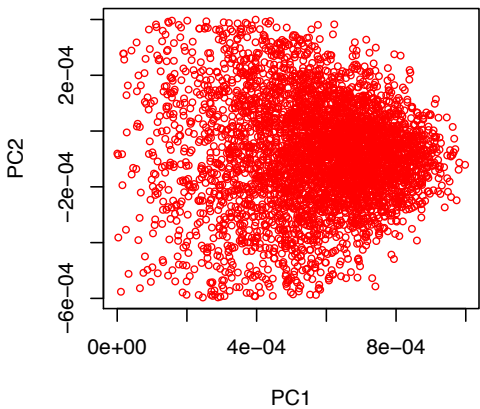

**D** Outliers removed

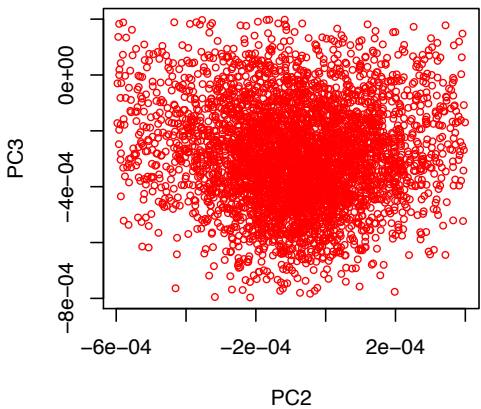

Supplement: S1 Fig — Principal component values of included subjects: (1) 0 ≤ PC1 ≤ 0.001, (2) -0.0006 ≤ PC2 ≤ 0.0004, (3) -0.0008 ≤ PC2 ≤ 0.0002. (PDF) [file pone.0187644.s001.pdf]

**A**

Raw samples of phs000091

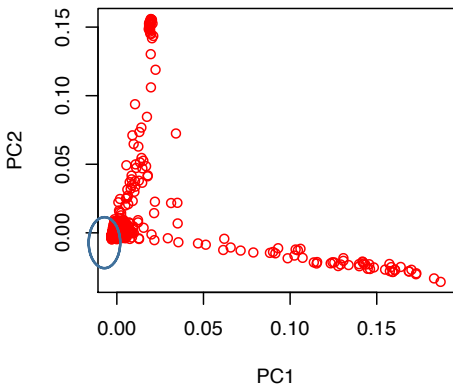**B**

Raw samples of phs000091

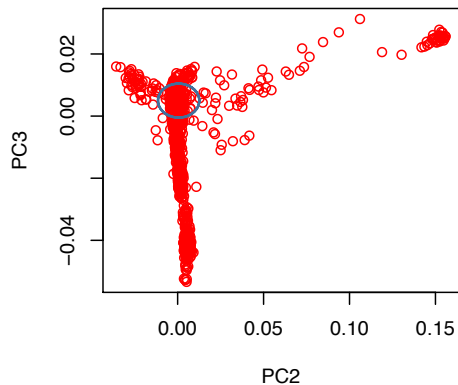**C**

Outliers removed

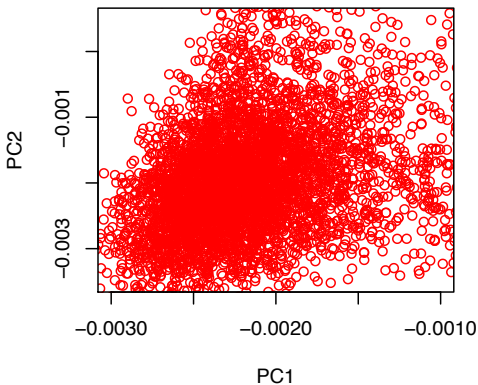**D**

Outliers removed

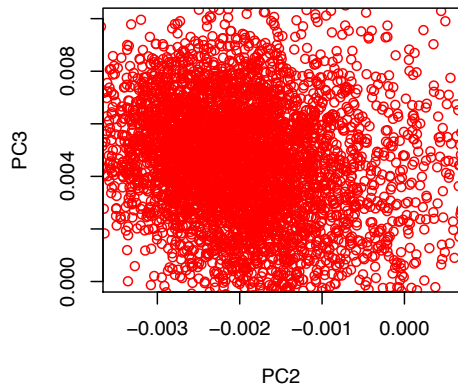

Supplement: S2 Fig — Principal component values of included subjects: (1) -0.003 ≤ PC1 ≤ -0.001, (2) -0.0035 ≤ PC2 ≤ 0.0005, (3) 0 ≤ PC3 ≤ 0.01. (PDF) [file pone.0187644.s002.pdf]
